# Clozapine clinics in north Wales - service evaluation audit

**DOI:** 10.1192/bjo.2021.900

**Published:** 2021-06-18

**Authors:** Jawad Raja, Zeenish Azhar, Masood Malik

**Affiliations:** 1 ST5 Betsi Cadwaladr University Health Board; 2 CT2 Betsi Cadwaladr University Health Board; 3 Clinical Director Betsi Cadwaladr University Health Board

## Abstract

**Aims:**

1. Quality of clozapine clinic appointment
2. Effectiveness of clozapine clinic servive
3. Compliance with BCUHB guidelines for physical health monitoring in clozapine clinics
4. We retrospectively audited 40 case notes 10 each from 4 differtent CMHT clozapine clinics

My role in the Project & How does this represent my practice?

1. I was the audit and overall lead for this project
2. I formulated the audit tool and registered my project with Audit Registration Team
3. I lead data collection and compilation of results

**Background:**

1. This audit followed up from a Coroner's investigation for a clozapine clinic patient
2. Clozapine is used for Treatment Resistant Schizophrenia but needs close monitoring due to potentially fatal side effects
3. NICE recommends annual monitoring of weight, blood pressure, waist measurement, blood glucose and plasma lipid levels

**Method:**

1. Has the patient been seen in the past year by clinician to monitor response to clozapine treatment?
2. Has the clozapine plasma level been measured during the last year of treatment?
3. Is brief MSE & Risk assessment documented during review?
4. Has Life style modification advice been provided?
5. Has annual physical health been completed?
6. Has Annual CTP/CPA been completed and documented?
7. Has the patient been allocated a named care coordinator?
8. Has clozapine side effects monitoring been documented?

**Conclusion:**

1. Clozapine is a superior medication for the treatment of refractory schizophrenia and is also be effective for other conditions
2. Clozapine is underused due to a variety of barriers related to the drug and its properties, the health care system & regulatory requirements
3. This service evaluation/quality improvement project provides the framework for clozapine clinics evaluation and recommends strategies for improvement

